# Ethical issues relating to renal transplantation from prediabetic living donor

**DOI:** 10.1186/1472-6939-15-45

**Published:** 2014-06-16

**Authors:** Aldo Ferreira-Hermosillo, Edith Valdez-Martínez, Miguel Bedolla

**Affiliations:** 1 Unidad de Investigación en Endocrinología Experimental, Hospital de Especialidades, Centro Médico Nacional Siglo XXI, Cuauhtémoc 330, colonia Doctores, 06729 Mexico City, Mexico; 2 Coordinación de Investigación en Salud, Centro Médico Nacional Siglo XXI, Cuauhtémoc 330, colonia Doctores, 06729 Mexico City, Mexico; 3 Policy Studies Center of the College of Public Policy, The University of Texas in San Antonio, 501 W. Cesar Chavez Blvd, San Antonio, TX 78207, USA

**Keywords:** Renal transplantation, Living donors, Organ donor, Prediabetes, Diabetes mellitus, Clinical ethic, Mexico

## Abstract

**Background:**

In Mexico, diabetes mellitus is the main cause of end − stage kidney disease, and some patients may be transplant candidates. Organ supply is limited because of cultural issues. And, there is a lack of standardized clinical guidelines regarding organ donation. These issues highlight the tension surrounding the fact that living donors are being selected despite being prediabetic. This article presents, examines and discusses using the principles of non-maleficience, autonomy, justice and the constitutionally guaranteed right to health, the ethical considerations that arise from considering a prediabetic person as a potential kidney donor.

**Discussion:**

Diabetes is an absolute contraindication for donating a kidney. However, the transplant protocols most frequently used in Mexico do not consider prediabetes as exclusion criteria. In prediabetic persons there are well known metabolic alterations that may compromise the long − term outcomes of the transplant if such donors are accepted. Even so, many of them are finally included because there are not enough donor candidates. Both, families and hospitals face the need to rapidly accept prediabetic donors before the clinical conditions of the recipient and the evolution of the disease exclude him/her as a transplant candidate; however, when using a kidney potentially damaged by prediabetes, neither the donor’s nor the recipient’s long term health is usually considered.

Considering the ethical implication as well as the clinical and epidemiological evidence, we conclude that prediabetic persons are not suitable candidates for kidney donation. This recommendation should be taken into consideration by Mexican health institutions who should rewrite their transplant protocols.

**Summary:**

We argue that the decision to use a kidney from a living donor known to be pre-diabetic or from those persons with family history of T2DM, obesity, hypertension, or renal failure, should be considered unethical in Mexico if the donor bases the decision to donate on socially acceptable norms rather than informed consent as understood in modern medicine.

## Background

The population of Mexico is estimated to be 123,278,559 [1]. Type 2 diabetes mellitus (T2DM) affects 6.4 million people [2], causing 40% of the cases of end-stage renal disease (ESRD). This condition requires treatment with dialysis (peritoneal or hemodialysis) or renal transplantation. The *Centro Nacional de Trasplantes,* (CENATRA, its acronym in Spanish, the agency responsible for the national transplant system) reports that kidneys are the most frequently requested organs in the country [3], while the donation rate is low (5 donors per million inhabitants) [4]. The most common form of renal transplantation in patients with-ESDR secondary to T2DM is from living donor (75% approximately), and the most common donors are first degree relatives (*i.e*.: parents, brothers, etc.) [5]. Donors therefore belong to the same risk population as the organ receptor; and they are selected despite their family history of T2DM or hypertension.

A person is diagnosed as prediabetic when she/he presents impaired fasting glucose (IFG) or impaired glucose tolerance (IGT). The former is determined when fasting blood glucose levels are between 100 mg/dl and 125 mg/dl. The latter is determined when blood glucose levels are between 140 mg/dl and 199 mg/dl—after the oral glucose tolerance test (OGTT) [6]. There is a controversy about whether a pre-diabetic person may be considered as renal donor candidate [7]. This controversy stems from three fundamental facts: First, the prevailing notion that a prediabetic person is “healthy”, which may not be entirely true. According to the American Diabetes Association it is only necessary to lose 7% of weight and do 150 minutes of physical activity per week to control blood glucose levels [8,9]. This goal is seemingly “easy” to achieve, yet it may cause some physicians underestimate the potential risks of prediabetes. Second, the lack of scientific publications analysing the long − term outcomes of prediabetic kidney donors and the consequences of diminished renal tissue. Third, the Amsterdam forum establishes that T2DM patients should not be renal donors because they have a high risk of developing diabetic nephropathy; but it does not make a statement about considering people with prediabetes [10].

The overwhelming demand for kidneys exacerbates the controversy; hence, most medical centres accept prediabetic donors despite the risks in order to reduce the number of people on the waiting lists. This raises medical, legal and ethical concerns: is reducing the number of people on the waiting list all that matters? Due to known donor prognosis, should we worry only about the welfare of the organ recipient and not worry at all about the welfare of the donor? What about the implementation of the constitutionally guaranteed right to health?

This work intends to examine and discuss the ethical, legal, and medical considerations about kidney donor selection in Mexico with the idea that such selection is not only a technical − medical act that increases the number of donors and decreases the number of people in the waiting lists, but that also must be concerned about the long − term prognosis of the donor and the recipient and the ethics of such donations.

## Discussion

### Epidemiological evidence

#### The argument that prediabetes constitutes a risk factor for T2DM

Prediabetes is not only associated with the development of T2DM, it is possible that prediabetes, by itself, can induce renal failure. This was observed in a crude analysis of the data from 2,398 people in the Framingham Heart Study (Table 1) which demonstrates that there is a 65% risk of developing chronic kidney disease in persons with IFG and IGT in comparison with normal persons [11]. In the case of a donor, this risk increases because of the reduced renal mass [12].

**Table 1:** Studies linking glucose level with the development of kidney damage

**Author and publication year**	**Study type and follow − up period**	**Place and population of study**	**Form of valuation**	**Results**
Fehrman-Ekholm et al. 2001 [13]	Cohort study with a follow − up of 12 years (April 1964 − December 1995)	Sweden	Normal initial OGTT	Six developed T2DM
		348 relative living donors (93.5% inbreeding)		
Aroda et al. 2008 [8]	Review study. Information from the National Centre for Chronic Disease Prevention and Health Promotion (2008)	USA	Fasting plasma glucose levels and OGTT	Risk to develop T2DM:
		National Study		0.7% normoglicemic, and
				5 − 10% IFG and IGT
Nichols et al. 2007 [14]	Cohort studies with a follow time of nine years (January 1994 − December 2003)	USA	Fasting plasma glucose levels between	From people with glucose levels between 100–109 mg/dL, 8.1% developed T2DM.
		5,452 members from the Kaiser Permanente Northwest		
				From people with glucose levels between 110–125 mg/dl, 24.3% developed T2DM
			100–109 mg/dl and 110–125 mg/dl	
Fox et al.	Cohort studies.	USA	Fasting plasma glucose levels and OGTT	Risk of 65% to develop CKF on people with IFG and IGT in comparison with control group
Framingham (Follow − up)	Initial time: 1991–1995 Follow − up period: 1998 − 2001	2,398 persons		
2005 [11]				
Azar et al. 2007 [15]	Cohort study with a follow − up of three years	Iran	Clinical and biochemical record	55% presented hypertension.
		Tabriz Medical Sciences University		
				7% Increased creatinine concentrations
		86 living donors, no related		10% presented severe depression

CKF = Chronic Kidney Failure.

A systematic review with data from 20 studies that included an overall total of 95,783 people, with a median of follow − up of 12.4 years (4–19 years), show that IFG and IGT constitute a risk factor to cardiovascular events (RR = 1.33 and 1.58 respectively) [16]. The same was observed in the DECODE study (Diabetes Epidemiology: Collaborative Analysis of Diagnostic Criteria in Europe) where prediabetes increased mortality rate [17]. A meta-analysis that included data from 10 randomized clinical trials, and an overall total 23,152 people, shows that treatment for IFG and IGT with diet, physical exercise or pharmacological therapy diminish the incidence of stroke and decrease the frequency of myocardial infarction in comparison with control groups [18] thereby showing that prediabetes poses an increase in risk for these events.

Unilateral nephropathy increases 70% the glomerular filtration rate and blood flow in the healthy kidney. These changes, in the long − term, can end − up in renal failure. This was demonstrated by Azar *et al.* (Table 1) on a study where 55% of renal donors presented complications, 7% had an increase in creatinine levels and 10% developed severe depression [15].

Moreover, the CARI guidelines (Caring for Australasians with Renal Impairment), a set of periodically updated guidelines strictly based on available evidence, included the results from 11 studies in which renal donors − without previously known risk factors − were monitored over 20 years [19]. The CARI guidelines report a global incidence of T2DM ranking between 1.5% and 1.7%; and conclude that despite absence of prediabetes, kidney donors could develop T2DM.

#### The argument that prediabetes does not constitute a risk factor for T2DM

While previous studies demonstrate the importance of excluding prediabetic persons as potential kidney donors, other reports show a low incidence of T2DM among prediabetic donors. This discussion recently re − emerged in the ‘Kidney Week’, held in San Diego, California, in December 2012. In the meeting of the American Society of Nephrology held during that week, Chandran presented the results from a retrospective cohort study performed at the University of California, San Francisco, USA. He monitored 35 renal donors with fasting glycemic levels >109 mg/dl for a 10 − year period. He found that 31% of the donors persisted with IFG, 11% developed T2DM, and 58% presented normal fasting glycemic levels [20]. These results favour the notion that not all prediabetic individuals develop T2DM; hence, many doctors widen their selection criteria of kidney donors based on these findings. However, Chandran sets aside the remaining 42% of prediabetic donors that continued to have impaired glucose regulation, who could present nephropathy at long − term. Chandran’s findings are consistent with the reported by Faerch et al. who show that 30% of people with prediabetes will not progress to T2DM [21]. A critical appraisal of this statement should be done as in the Mexican population exist conditions that increase the risk of such progression [22]; e.g.: family history of diabetes mellitus, obesity, sedentary lifestyle, hypertension, gestational diabetes, and polycystic ovary syndrome in women.

As to the impact of nephrectomy, there are also some publications that report that it is not associated with impaired renal function or the development of other comorbidities. For example, Narkun-Burgess *et al.* report 62 young men that underwent a nephrectomy secondary to trauma during World War II. 28 out of 62 men died before 1993 and only six suffered changes in renal function that were not related to the nephrectomy. In the survivor group, five had macroproteinuria and three had serum creatinine levels higher than 1.5 mg/dl. The authors conclude that after a 45 − year follow − up; nephrectomy did not contribute to renal failure nor increase mortality among the subjects. It is important to highlight that there are no prospective longitudinal studies that evaluate the prognosis of prediabetic persons who undergo a nephrectomy [23].

Therefore, the evidence is not conclusive. It seems that the stronger epidemiological evidence shows that there exists a high risk of developing T2DM in kidney donors than the available evidence in favour of kidney transplantation from living donors. But the uncertainty remains. Nonetheless, the expected benefits for the renal recipient along with the decrease in the number of people waiting lists are tempting doctors to lean towards renal transplantation. Which is the ethical decision?

#### The argument from a Mexican perspective

Most of the studies that explore the controversy in authorizing or rejecting donation from prediabetic people have been carried out among Caucasian populations. Thus, in order to answer the ethical question it is necessary to consider the fact that the Mexican population is a Mestizo population, product of the intermarriage of Caucasians and Native Americans, and as a result it has significantly different genotypic and cultural characteristics, and they have to be considered in the decision − making process regarding organ donation in Mexico.

Genetically, Mexicans seem to be at higher risk for developing T2DM. Studies show that the population has high frequencies of polymorphisms affecting some of the genes involved in glucose and lipid metabolism. One of them is the polymorphism on the *HNF4A* gene, which is involved on insulin regulation and has a high frequency among the Mexican mestizo population. Another polymorphism is the variant R230C from the *ABCA1* gene, one of the main risk alleles found on Amerindian or Amerindian-derived populations [24]. Furthermore, 60% of the Mexican population has a family history of diabetes. This family background is associated with an increase in fasting insulin levels (OR 1.7), a decrease in insulin sensibility (OR 1.95), and an increase in the risk of diabetes (OR 1.63) [25].

Regarding cultural characteristics, Mexico is a country where the diet has been adversely modified recently by the introduction of so − called “fast foods” and the increasing consumption of sugar based beverages. Batis *et al.* found that Mexicans’ dietary habits had become less healthy by 2006, when they compared the changes in the Mexican diet between 1999 (low U.S. influence) and 2006 (high U.S. influence) [26]. Likewise, Fanghälen-Salmón *et al.* report that the prevalence of a sedentary lifestyle is of almost 65 − 80% in the Mexican population [27]. And the Mexican population does not easily accept the lifestyle modifications that have to be made to treat prediabetes [28]. Thus, it is expected that most of the Mexican prediabetics will eventually become diabetic.

In order to detect chronic kidney disease among the Mexican adult population, a pilot study was made by the National Kidney Foundation: Kidney Early Evaluation Program (KEEP). This study (performed in 2008) included participants with T2DM and HTN with chronic kidney disease. The survey required that the patients completed a questionnaire and provided a blood and urine sample. The results showed a high prevalence of chronic kidney disease among participants with T2DM (38%), and with T2DM and HTN (42%). It should be noted that most of the participants ignored their health condition despite the fact that 71% of them had visited their primary care physician in the previous year [29].

#### The legal argument: the unclear laws for organ donation in Mexico

The Mexican General Law of Health [30] in Title 14, section 333, establishes that the requirements for transplantation from living donors are: to be older than 18 years of age and in full possession of their mental faculties, to donate organs with a function that can be compensated by another organ, be compatible with the receptor, to receive complete information about the procedure beforehand, to give informed consent and to have a family bond with the receptor. The law does not consider any comorbidity as a restriction for donation.

Section 313 of the same law mentions that the Ministry of Health is responsible for monitoring health issues during transplantation of organs, tissues, and cells of human beings through the CENATRA. This agency establishes that “medical and legal constraints will be analyzed in each particular case by the internal transplant committee” and that “to judge whether medical limitations exist, health professionals must perform a detailed bioethical, medical and legal assessment of the donor to eliminate sanitary risk to the receptor, as well as to assure that the organ and/or tissue be in acceptable condition to meet the receptor’s needs”. It is noteworthy to mention that sections 326 and 332 of the same General Law of Health prohibit the donation from minors (except for bone marrow), the mentally incompetent, and pregnant women [30]. Again, the CENATRA’s guidelines do not specify the legal aspects regarding comorbidities in the kidney donor.

The World Health Organization issued guidelines on cell, tissue, and organ transplantation. Paragraph 10 states that: “High-quality, safe and efficacious procedures are essential for donors and recipients alike. The long − term outcomes of cell, tissue and organ donation and transplantation should be assessed for the living donor as well as the recipient in order to document benefit and harm.”

When the Mexican Law and the World Health Organization’s statement are compared it is obvious that the Mexican laws and action plans are focused on the receptor’s well − being. The Mexican law does not establish a legal framework to identify donor comorbidities that should contraindicate donation. In countries like Mexico, with substantially high rates of T2DM, obesity, hypertension, and renal failure, and limited access to medical care, the ethical issues regarding renal transplant require a unique “national” approach in order to improve social and moral regulations.

#### The argument from non − maleficience

Accepting a kidney from a Mexican living − donor with prediabetes represents a risk for the donor, and it also raises a question about whether the ethical principle of *primum non nocere* (“first do no harm”) is being respected [31]. The authors consider that sometimes the guidance and authority of this ethical principle may be neglected during the process of making the decision to transplant a kidney from a prediabetic donor, especially when it is a family member who needs the organ. It is necessary to distinguish between donor well − being, and benefit to others. Doctors have the moral obligation of exercising due care, balancing intended benefits against risks and inevitabilities of harm, physical, psychological, and social. Primum non nocere, non-maleficience as it is now called, should guide physicians to protect potential donors from harming themselves, including prediabetics who “voluntarily” decide to accept kidney donation for a family member or others. If there is a possibility of injury to the donor, it might be then considered that the recipient continues to be with peritoneal dialysis or hemodialysis until a new donor without comorbidities is located.

#### The argument from respect for person’s autonomy

It’s not easy to avoid the fact that society, health institutions and family exert pressure for organ donation. For potential donors, it is not easy to dispassionately consider themselves as obligated donors. The decision to donate a kidney to a family member, or friend, could be made under a form of coercion that is socially accepted and yet it denies the donor of the effective freedom to consent. The potential donor may also be under the effect of an undue influence to meet family values or the urgency of the transplant team. However, respect for a person’s autonomy and their right to freely consent or refuse an intervention, are core values of modern medicine. The autonomy of a person is respected when the risk and possible benefits of the donation are presented and discussed before the potential donor makes a decision. Although doctors cannot assure of each and every one of the risks and benefits of donation, because each individual is unique, transplant specialists must distinguish socially acceptable decisions from ethical decisions. In Mexico, a number of ethical questions immediately arise from this question: can we talk about respect for autonomy (the ability to take decisions freely without any coercion or undue influence) when consent is given because it is socially expected or when the risk and benefits of the donation have not been fully explained?

Some health professionals believe that informed consent releases them from the responsibility of protecting donors because they “knowingly and voluntarily” accept the potential damage to their health. This belief is in direct conflict with section four of the Mexican Constitution, which states that every Mexican citizen has a right to health and by implication a right to the decision that is in the best interest of his/her health [32]. In fact, informed consent must be a tool for donors and receptors to assess potential risks and it must be designed to protect both of them equally.

#### The argument that prediabetic living donor would be incompatible with the ideal of justice

Justice requires treating people fairly, according to their individual needs and merits [31]. A prediabetic has high probabilities of develop T2DM and eventually renal failure, thus, it is difficult to see that he/she needs or merits to be considered for a kidney donation. Even so, it could be difficult to see why renal transplantation from prediabetic donor is inherently wrong, especially if the person is fully aware of his/her own condition and its risks and honestly believe that the donated kidney will improve quality of life for a close relative or friend. It could be an example of love. Such a potential donor is moved by care and concern for the relative that will benefit from the donation. The donor’s right to health is at issue. However, in modern society there are many instances in which people surrender their rights, even the right to life, in order to benefit others. Such is the case of people in the Military and in law enforcement. It may happen that a good purpose is served; but the notion of human rights is a notion that places limits on how an individual may be treated, regardless of the good purposes that might be accomplished.

## Summary

There is an unmet demand for kidney for transplantation, yet despite the obvious need, this is not sufficient justification for the use of prediabetic living donors. Although epidemiologically there are arguments for and against, sound prospective longitudinal studies that evaluate the prognosis of prediabetic persons are unavailable at the present time. However, there are stronger epidemiological evidence and typical aspects of the Mexican situation showing that there exists a high risk of developing T2DM in prediabetic living donor than the available evidence in favour of renal transplantation. Ethical concerns emerge when donations from prediabetic persons are analysed from the point of view of the ethical principles of non − maleficience, autonomy and justice. The doctors’ obligation to do no harm to their patients would seem imply that when conflict arises between benefiting a patient that requires renal transplantation and benefit a potential organ donor then *prima facie* the potential donor’s interests should take priority.

## Abbreviations

IFG: Impaired fasting glucose; IGT: Impaired glucose tolerance; OGTT: Oral glucose tolerance test; T2DM: Type 2 diabetes mellitus; ESRD: End − stage renal disease.

## Competing interests

The authors declare that they have no competing interests.

## Authors’ contributions

AF made substantial contribution to conception, design, acquisition and interpretation of the references; and has been involved in drafting the manuscript. EV contributed to conception and design; and has been involved in drafting the manuscript and revising it critically for intellectual content. MB has been involved in drafting the manuscript and revising it critically for intellectual content and consistency. All authors read and approved the final manuscript.

## Authors’ information

Aldo Ferreira is an endocrinologist. His clinical and research interests are focused on diabetes mellitus, obesity and metabolic syndrome. Edith Valdez, M.D., MSc, DrPH. She is a medical researcher. Her current areas of interest include medical ethics, research ethics, and clinical epidemiology. Miguel Bedolla, BA, MD, PhD, MPH is a researcher and medical ethics consultant. His current areas of interest include the philosophical foundations of medical ethics, research ethics and military and law enforcement ethics.

## Pre-publication history

The pre-publication history for this paper can be accessed here:

http://www.biomedcentral.com/1472-6939/15/45/prepub
